# Prostate-specific antigen response and clinical progression-free survival in Black and White men with chemotherapy-naïve metastatic castration-resistant prostate cancer treated with enzalutamide in a real-world setting

**DOI:** 10.1038/s41391-022-00622-6

**Published:** 2022-12-14

**Authors:** Stephen J. Freedland, Agnes Hong, Nader El-Chaar, Sharanya Murty, Krishnan Ramaswamy, Anna D. Coutinho, David Nimke, Alicia K. Morgans

**Affiliations:** 1 Center for Integrated Research in Cancer and Lifestyle, Cedars-Sinai Medical Center, Los Angeles, CA USA; 2 Durham VA Health Care System, Durham, NC USA; 3 Astellas Pharma Inc., Northbrook, IL USA; 4 Xcenda, Carrollton, TX USA; 5 Pfizer Inc., New York, NY USA; 6 Dana-Farber Cancer Institute, Boston, MA USA

**Keywords:** Prostate cancer, Outcomes research

## Abstract

**Background:**

In the United States, Black men have a higher incidence of prostate cancer (PC)-related mortality than men of other races. Several real-world studies in advanced PC suggest, however, that Black men respond better to novel hormonal therapies than White men. Data on treatment responses to enzalutamide by race are limited. We assessed real-world prostate-specific antigen (PSA) response and clinical progression-free survival (cPFS) of Black vs. White men with chemotherapy-naïve PC treated with enzalutamide.

**Methods:**

This retrospective cohort study included patients with PC who initiated enzalutamide treatment from 2014 to 2018 in the IntrinsiQ Specialty Solutions™ database, a collection of electronic medical records from community urology practices. Index date was the date of the first prescription for enzalutamide, used as a proxy for metastatic castration-resistant PC (mCRPC). Patients who had undergone chemotherapy and/or abiraterone therapy were excluded. Kaplan–Meier and Cox models adjusted for baseline characteristics were used to estimate PSA response and cPFS by race.

**Results:**

The study included 214 Black and 1332 White men with chemotherapy-naïve PC presumed to have mCRPC based on the enzalutamide indication during the study period. Black men were younger and had higher baseline median PSA levels than White men. Enzalutamide therapy duration, follow-up time, and number of post-index PSA tests were similar between races. In multivariable analyses, the risk of patients achieving a ≥ 50% PSA decline was similar, whereas a numerically higher trend of ≥90% PSA decline was observed in Black men (HR 1.23; 95% CI 0.93–1.62 [*P* = 0.14]). In the multivariable analysis, Black men had significantly better cPFS (HR 0.82; 95% CI 0.68–0.98 [*P* = 0.03]).

**Conclusions:**

Black and White men with presumed chemotherapy-naïve mCRPC had similar PSA responses when treated with enzalutamide, but Black men had better cPFS than White men. Further research is warranted to validate these findings.

## Introduction

In the United States (US), Black men are 1.75 times more likely to be diagnosed with and twice as likely to die of prostate cancer (PC) than White men [1]. Disparities in the incidence and mortality rate of PC and in screening and access to treatment between Black and White men are well documented [1–5]. The racial disparities in PC are likely multifactorial, including social, cultural, and biological determinants of health [3, 6–8]. In addition, Black men are under-represented in randomized controlled trials and prospective observational studies [9, 10].

The treatment landscape for chemotherapy-naïve metastatic castration-resistant PC (mCRPC) has evolved in the past decade. The emergence of novel hormone therapies (NHTs), including enzalutamide, has substantially improved overall survival (OS) in this disease setting [11–17]. There is insufficient robust clinical efficacy data by race of prostate-specific antigen (PSA) responses and other treatment outcomes, including progression and survival, from clinical trials among patients with chemotherapy-naïve mCRPC.

Early PSA response with NHTs in mCRPC is an independent prognostic factor for survival [18, 19]. Real-world studies evaluating patients with mCRPC focusing on PSA outcomes suggest that Black men have better PSA responses and survival outcomes than White men when treated with NHTs such as enzalutamide and abiraterone [20–23]. Most of these studies, however, are conducted by single institutions, include patients with prior exposure to chemotherapy for mCPRC, or are relatively small [21–23]. This study aims to understand the differences in PSA treatment responses and clinical progression by race in a real-world population of patients (in a community urology electronic health record [EHR] database) with chemotherapy-naïve PC treated with enzalutamide, at a time when enzalutamide was approved only for the treatment of mCRPC. Based on prior literature results, we hypothesized that PSA outcomes in Black men would be similar to or better than those in White men.

## Methods

### Study design and data source

This was an observational, retrospective cohort study conducted on data from patients with chemotherapy-naïve PC in the IntrinsiQ Specialty Solutions™ (IQSS) urology electronic medical records (EMRs) database. IQSS data from 2015 include aggregated data of all patients (benign prostatic hyperplasia, bladder cancer, erectile dysfunction, overactive bladder, PC, and stress urinary incontinence) from ~30% of independent community urologists in the US. The database included ~2.1 million active patients in 2018.

The study period was from September 1, 2013, to June 30, 2018, and the identification period was from September 1, 2014, to February 28, 2018. The index date was the date of the first prescription of enzalutamide during the identification period. Patients were observed for ≥12 months before the index date (baseline) to characterize this population before the initiation of enzalutamide treatment (pre-index period). The follow-up period was from the index date to the earliest date of one of the following events: observation of a study endpoint, death, last visit date, or end of study period. This study was exempt from internal review board approval since all assessed data were restricted to deidentified patient records.

### Study population

**Fig. 1:**
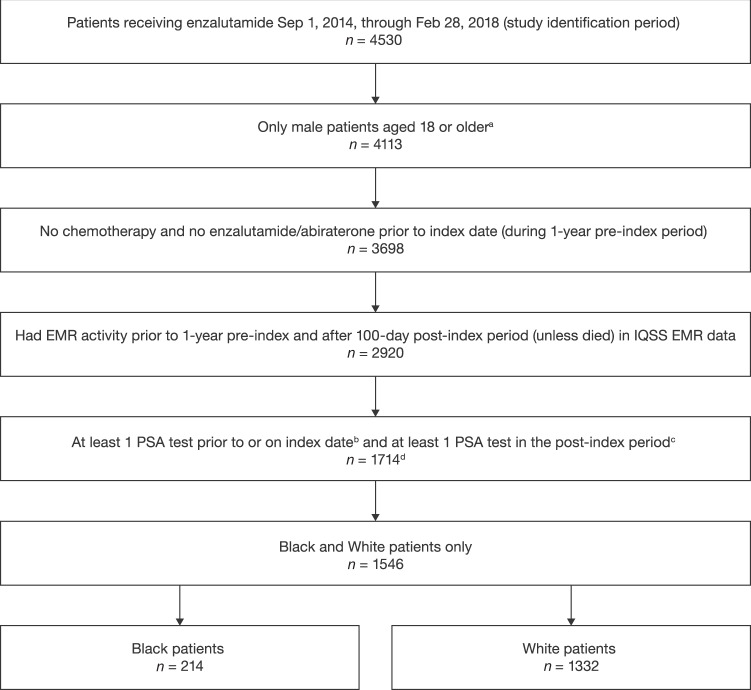
Patient attrition. ^a^Patients with missing age values were excluded; ^b^During 60 days pre-index; ^c^Unless died; ^d^168 patients did not fall under these two race categories or had unreported race information and were excluded from this analysis (1546 total patients compared). EMR electronic medical record, IQSS IntrinsiQ Specialty Solutions^TM^, PSA prostate-specific antigen.

The study population included adult (aged ≥ 18 years) Black and White (recorded race in the EMR) male patients with chemotherapy-naïve PC treated with enzalutamide (see Fig. 1 for full selection criteria). As enzalutamide was approved exclusively for the treatment of mCRPC at this time, enzalutamide usage without evidence of having undergone chemotherapy and/or abiraterone therapy was used as a proxy for first-line treatment of mCRPC. Patients must have had ≥1 PSA test within 60 days before or on the index date and ≥1 PSA test during follow-up, except those who died during follow-up, thereby avoiding survival bias. Patients who met the sample selection criteria were categorized in cohorts according to their recorded race in the EMR (Black vs. White). Patients not categorized by these two races or who had unreported race information were excluded.

### Study measures

#### Baseline patient demographics and clinical characteristics

Demographics included age, marital status, geographic region of urology practice, and year of enzalutamide index date. Clinical characteristics evaluated were baseline PSA level, documentation of (yes/no) and site(s) of metastasis, cardiovascular comorbid conditions, Charlson Comorbidity Index (CCI), and lab tests of interest (testosterone). Patients’ treatment history of hormone therapies, prostatectomy, radiation, use of bone-targeting agents, corticosteroid therapies, and pain management before the index date was also assessed.

#### PSA response and clinical progression-free survival (cPFS)

The primary outcome evaluated was PSA response, which compared the cumulative incidence of Black and White men who achieved a decline in PSA concentrations of ≥50%, ≥75%, and ≥90% and absolute PSA concentrations of <0.2 ng/mL, <0.1 ng/mL, and <0.01 ng/mL during follow-up. cPFS was a composite outcome based on the earliest occurrence of the following: (1) 25% increase or an absolute increase of ≥2 ng/mL above the nadir or above the baseline (if all post-baseline PSA values are higher than baseline); (2) switch to second-line treatment; or (3) all-cause death. The cPFS definition used in this study was a modification of the Prostate Cancer Working Group recommendations [24] since the study population included patients presumably in the last stage of PC and clinicians often switch to another agent before conducting a confirmatory PSA test. Reducing the number of required post-index PSA tests from three to one may prevent missing a progression event due to a lack of confirmatory PSA rise. Post-index follow-up measures, including duration of follow-up in the database, duration of enzalutamide treatment, and number of follow-up PSA tests, were also assessed.

### Statistical analysis

All baseline demographics and clinical characteristics were summarized using descriptive statistics by race. Continuous measures were described as means with SDs for normally distributed variables and medians with IQRs for other variables; binary and categorical variables were presented as percentages. Differences at baseline between the races were described by standardized mean differences (SMDs), and White men were considered the reference race. An SMD between Black and White men of >10% was considered unbalanced [25].

Kaplan–Meier curves were used to estimate the cumulative incidence of patients’ treatment responses during follow-up (PSA decline of ≥50%, ≥75%, and ≥90%; cPFS). In addition, multivariable Cox proportional hazard models were used to estimate HRs for PSA response and cPFS by race and were adjusted for baseline covariables of age, region, marital status, year of enzalutamide index date, CCI, baseline log PSA level, pre-index treatments, and documentation status (yes/no) of metastasis (since the data for the site of metastasis were limited, the data could not be included as a variable).

In the PREVAIL study, which included patients with chemotherapy-naïve mCRPC treated with enzalutamide or placebo, median time to PSA progression was 11.2 months [14]. Based on this result, we performed a 12-month landmark sensitivity analysis for cPFS to account for follow-up limitations within the urology database. Since the current study is in a urology setting, many patients will transition to oncology care, contributing to loss of follow-up within the urology EHR. In the sensitivity analysis, patient follow-up spanned from the initiation of enzalutamide therapy to the first occurrence of censored at last visit date, end of 12 months, or June 30, 2018, whichever occurred first.

## Results

### Patient characteristics

Of 1546 male patients, 214 (13.8%) were Black and 1332 (86.2%) were White (Table 1). Compared with White men, Black men were younger and had a higher incidence of mild-to-moderate diabetes (24.3% vs. 16.7%) and hypertension (60.3% vs. 51.4%). They were more likely to use pain management medication in the pre-index period (36.9% vs. 29.7%) and less likely to use bone-targeting agents (35.5% vs. 45.5%). In addition, Black men had a higher median baseline PSA level (17.6 ng/mL IQR [4.0–56.4] vs. 10.5 ng/mL IQR [3.2–37.7]) and more often had non-castrate testosterone levels >50 ng/dL (7.0% vs. 2.7%), though testosterone data were available only in 37.9% and 28.5% of Black and White men, respectively (Table 1).

**Table 1:** Baseline characteristics.

Characteristics/clinical outcomes	Black patients (*n* = 214)	White patients (*n* = 1332)	SMD (%)
Age, years, *n* (%)			44.0^a^
40–59	19 (8.9)	38 (2.9)	
60–79	145 (67.8)	746 (56.0)	
≥80	50 (23.4)	548 (41.1)	
Marital status, *n* (%)			51.5^a^
Patients with data	187 (87.4)	1214 (91.1)	
Single	41 (21.9)	99 (8.2)	
Married	107 (57.2)	952 (78.4)	
Divorced/separated	39 (20.9)	163 (13.4)	
Geographic region, *n* (%)			39.5^a^
Patients with data	213 (99.5)	1325 (99.5)	
Midwest	50 (23.5)	375 (28.3)	
Northeast	44 (20.7)	250 (18.9)	
South	112 (52.6)	549 (41.4)	
West	7 (3.3)	151 (11.4)	
Year of index therapy initiation, *n* (%)			22.4^a^
2014	22 (10.3)	94 (7.1)	
2015	69 (32.2)	365 (27.4)	
2016	54 (25.2)	450 (33.8)	
2017	63 (29.4)	383 (28.8)	
2018	6 (2.8)	40 (3.0)	
Comorbidities			
CCI, mean (SD)	0.78 (1.07)	0.84 (1.11)	–5.4
CCI comorbidities any time before the index date, *n* (%)^b^			
Any tumor, including leukemia and lymphoma	25 (11.7)	246 (18.5)	–19.1
Diabetes (mild to moderate)	52 (24.3)	222 (16.7)	19.0^a^
Myocardial infarction	5 (2.3)	75 (5.6)	–16.9
Rheumatologic heart disease and fever	0 (0.0)	10 (0.8)	–12.3
Comorbid conditions of interest any time before the index date, *n* (%)^b^			
Hypertension	129 (60.3)	685 (51.4)	17.9^a^
Ischemic heart disease	3 (1.4)	67 (5.0)	–20.7
Treatment history, *n* (%)			
Prostate cancer–specific therapy any time before the index date^b^	211 (98.6)	1302 (97.7)	6.4
First-generation antiandrogen therapy any time before the index date	152 (71.0)	884 (66.4)	10.1^a^
Hormone therapy any time before the index date	203 (94.9)	1243 (93.3)	6.5
Radiation therapy	39 (18.2)	293 (22.0)	–9.4
Radical prostatectomy	51 (23.8)	365 (27.4)	–8.2
Pain management	79 (36.9)	396 (29.7)	15.3^a^
Bone-targeting agents	76 (35.5)	606 (45.5)	–20.4
Clinical characteristics			
Site of metastasis			
Undocumented site of metastasis, *n* (%)	151 (70.6)	913 (68.5)	4.4
Documented site of metastasis, *n* (%)	63 (29.4)	419 (31.5)	
Baseline PSA, ng/mL, median (IQR)	17.6 (4.0–56.4)	10.5 (3.2–37.7)	18.0^a^
Testosterone, ng/dL			
Patients with data for testosterone, *n* (%)	81 (37.9)	380 (28.5)	
Median, *n* (IQR)	26.2 (17.3–43.0)	20.0 (12.0–31.0)	
Patients with testosterone >50 ng/dL, *n* (%)	15 (7.0)	36 (2.7)	
Median, *n* (IQR)	76.0 (56.0–208.0)	99.5 (64.5–305.5)	

*CCI* Charlson Comorbidity Index, *IQR* interquartile range, *PSA* prostate-specific antigen, *SMD* standardized mean difference.

^a^SMDs between Black and White men >10% are considered unbalanced. SMDs were analyzed among patients only with available data.

^b^The pre-index period includes the enzalutamide index date.

### Patient follow-up

Several measures, such as differences in median follow-up and number of post-index PSA tests, were assessed by race to evaluate whether they influenced the surveillance-based outcomes. Median duration of enzalutamide therapy [IQR] (Black = 10.9 months [6.0−16.9]; White = 10.3 months [5.6–16.3]), median post-index follow-up time (Black = 19.3 months [9.6−31.6]; White = 18.6 months [10.5−28.6]), and median number of post-index PSA tests [IQR] (Black = 2 [1.0−5.0]; White = 3 [1.0–5.0]) (Supplementary Fig. 1) were similar between the races in the study sample.

### PSA response

Despite the higher median baseline PSA levels in the Black men, a similar cumulative incidence of Black and White men achieved PSA concentrations <0.2 ng/mL (Black men: 30.3% vs. White men: 22.9%; *P* = 0.810), <0.1 ng/mL (Black men: 13.6% vs. White men: 19.8%; *P* = 0.824), and <0.01 ng/mL (Black men: 4.4% vs. White men: 1.6%; *P* = 0.797) by the end of the study (Table 2). The cumulative incidence of Black men compared with White men who achieved a PSA response was as follows: ≥50% (Black men: 63.1% vs. White men: 64.3%; *P* = 0.365), ≥75% (Black men: 49.9% vs. White men: 51.4%; *P* = 0.403), and ≥90% PSA decline (Black men: 38.9% vs. White men: 34.2%; *P* = 0.048]) (Table 2).

**Table 2:** PSA response outcomes.

PSA response outcome		Black patients	White patients	*P* value
		(*n* = 214)	(*n* = 1332)	
≥50% decline	*n* (%)	119 (55.6)	686 (51.5)	
	KM adjusted rate (%)	63.1	64.3	0.3648
	Unadjusted Cox analysis, HR (95% CI)	1.09 (0.90–1.33)	Ref	0.3660
	Multivariable Cox analysis, HR (95% CI)	1.02 (0.83–1.25)	Ref	0.8822
≥75% decline	*n* (%)	91 (42.5)	517 (38.8)	
	KM adjusted rate (%)	49.9	51.4	0.4029
	Unadjusted Cox analysis, HR (95% CI)	1.10 (0.88–1.37)	Ref	0.4037
	Multivariable Cox analysis, HR (95% CI)	1.04 (0.82–1.31)	Ref	0.7638
≥90% decline	*n* (%)	67 (31.3)	329 (24.7)	
	KM adjusted rate (%)	38.9	34.2	0.0484
	Unadjusted Cox analysis, HR (95% CI)	1.30 (1.00–1.69)	Ref	0.0490
	Multivariable Cox analysis, HR (95% CI)	1.23 (0.93–1.62)	Ref	0.1435
<0.2 ng/mL PSA level	*n* (%)	36 (16.8)	206 (15.5)	
	KM adjusted rate (%)	30.3	22.9	0.8096
<0.1 ng/mL PSA level	*n* (%)	18 (8.4)	112 (8.4)	
	KM adjusted rate (%)	13.6	19.8	0.8244
<0.01 ng/mL PSA level	*n* (%)	3 (1.4)	15 (1.1)	
	KM adjusted rate (%)	4.4	1.6	0.7966

*KM* Kaplan–Meier, *PSA* prostate-specific antigen, *Ref* reference.

**Fig. 2:**
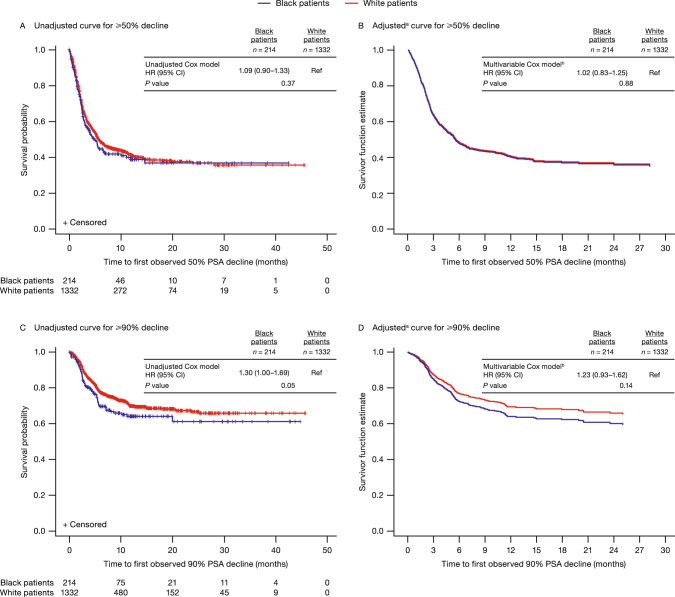
Kaplan–Meier curves for PSA decline. ^a^Since adjusted curves graph only events, the population is followed up only until the last event. Any values censored after the last event cannot be displayed on adjusted KM curves. ^b^Multivariable Cox proportional HR adjusted for baseline covariates of age, region, marital status, year of enzalutamide treatment initiation, CCI, baseline log PSA, pre-index treatments, and documentation of metastasis. CCI Charlson Comorbidity Index, KM Kaplan–Meier, PSA prostate-specific antigen, Ref reference.

In the unadjusted and multivariable analyses, the risk of achieving a ≥ 50% PSA decline (Fig. 2A, B) and ≥75% PSA decline (Supplementary Fig. 2) was similar between Black and White men. Black men trended toward having a greater risk (HR 1.23; 95% CI 0.93–1.62) of reaching a ≥ 90% decline in PSA levels (Fig. 2C, D) than White men, though this difference was not statistically significant in the multivariable model. In the multivariable analyses, baseline PSA level was the only variable associated with all three measures of a PSA response as shown in Supplementary Table 1.

### cPFS outcomes

**Fig. 3:**
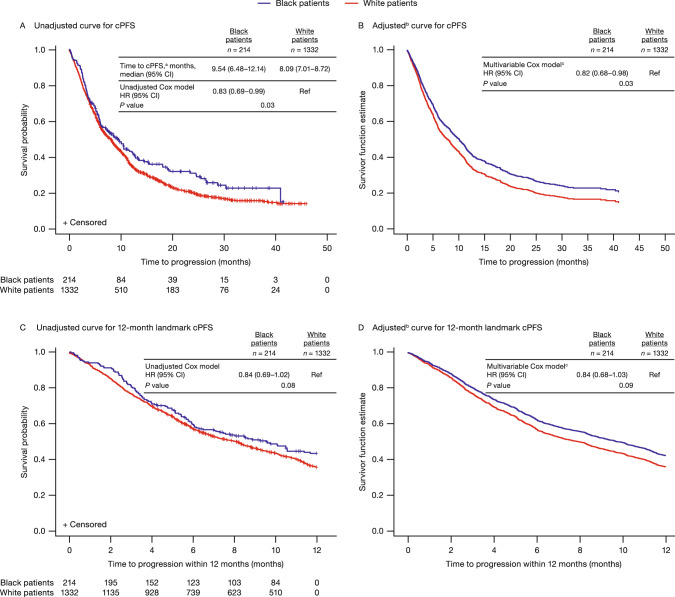
Kaplan–Meier curves for cPFS. ^a^cPFS was defined as the earliest of the following: ≥25% increase or an absolute increase of ≥2 ng/mL above the baseline (if all post-baseline PSA values were higher than the baseline) or the nadir; change to second-line treatment; or all-cause death; ^b^Since adjusted curves graph only events, the population is followed up only until the last event. Any values censored after the last event cannot be displayed on adjusted KM curves. ^c^Multivariable Cox proportional HR adjusted for baseline covariates of age, region, marital status, year of enzalutamide treatment initiation, CCI, baseline log PSA, pre-index treatments, and documentation of metastasis. CCI Charlson Comorbidity Index, cPFS clinical progression-free survival, KM Kaplan–Meier, PSA prostate-specific antigen, Ref reference.

Median duration to clinical progression was longer for Black men (9.5 months [95% CI 6.5–12.1]) than White men (8.1 months [95% CI 7.0–8.7]). In the multivariable model, Black men had a reduced risk of clinical progression (HR 0.82; 95% CI 0.68–0.98) compared with White men (Fig. 3A, B). In this multivariable analysis, baseline PSA level and receiving pain management were some of the factors associated with cPFS as shown in Supplementary Table 2.

### Sensitivity analysis

The 12-month landmark cPFS analysis revealed a 16% reduced risk of clinical progression among Black men in the multivariable model; however, the outcomes were not statistically significant (HR 0.84; 95% CI 0.68–1.03) (Fig. 3C, D). Analysis of other factors associated with progression is shown in Supplementary Table 3.

## Discussion

This study is the first large observational, retrospective cohort study conducted in the US urology setting that compared PSA response and cPFS treatment outcomes among Black and White men with presumed chemotherapy-naïve mCRPC treated with enzalutamide. The study results suggest that the PSA response outcomes measured as PSA decline (≥50%, ≥75%, and ≥90%) and reduced PSA concentration (<0.2 ng/mL, <0.1 ng/mL, and <0.01 ng/mL) during enzalutamide treatment were similar between races. cPFS was favorable among Black men, who had a significantly reduced risk (HR 0.82; 95% CI 0.68–0.98) compared with White men. These results suggest that Black men have a similar PSA decline when treated with enzalutamide but perhaps have a longer duration of clinical response than White men.

A growing body of evidence suggests the clinical benefit of enzalutamide for the treatment of advanced PC in Black and White men [20, 21, 26]. A few studies have shown that Black men tend to have a more significant PSA response (≥50% PSA level decline) than White men when treated with NHTs [20, 21]. In this study, Black men with chemotherapy-naïve PC had improved clinical survival outcomes (cPFS) compared with White men, a result that is consistent with previous evidence showing that Black men with mCRPC have a reduced risk of progression and better OS when treated with NHTs and other life-prolonging therapies [20, 26, 27]. These prior studies on NHTs combined data from enzalutamide plus abiraterone or analyzed abiraterone alone, unlike the current study, which focused only on enzalutamide and addressed the limited amount of real-world PSA response literature for enzalutamide treatment. In line with the longer time to cPFS in Black men than White men in this study (9.5 months vs. 8.1 months), a prospective study reported that time to PSA progression was better in Black men (16.6 months) than White men (11.5 months) with mCRPC treated with NHTs [20]. Although the trends observed in this prior study are like those in our study, the difference in observed PFS is attributable to the retrospective nature of our study and the differences in the endpoint we assessed. Our study evaluated cPFS, a more conservative endpoint than PSA-PFS, which focuses only on the increase in PSA. cPFS as a composite endpoint has been used previously as one of the endpoints to assess the efficacy of enzalutamide in patients with metastatic PC [28] Another study, which had a median follow-up of 19 months, showed that Black men with mCRPC had a significantly lower risk of death than White men, with a 33% risk reduction when treated with NHTs (either abiraterone or enzalutamide as first-line therapy) [26]. Conversely, a recent study using the community oncology EHR Flatiron Health database with a short median follow-up duration (13 months) showed that although treatment with both NHTs was associated with a similar median OS in White men and Black men (24 months), enzalutamide may have a marked superiority over abiraterone in increasing OS in White men (median OS: 20 months [enzalutamide] vs. 17 months [abiraterone]; HR of death 1.21; 95% CI 1.06–1.38) [27]. The difference in outcomes observed in the latter two studies may be attributable to the inherent strengths and limitations of the study designs and the analyzed data source [26, 27]. Further, the 12-month landmark cPFS analysis was performed in this study to assess early trends in treatment outcome and to address the challenge of non-availability of data for the entire follow-up period due to movement of patients to an oncology setting. This analysis reported a 16% lower risk in Black men, which was statistically insignificant. However, it is important to further evaluate the comparative effectiveness of the two NHTs in men of both races to better characterize these differences, if they exist.

The limitations of this study warrant discussion. The population included in this study was presumed to be patients with chemotherapy-naïve mCRPC as we excluded patients with prior chemotherapy exposure and at the time of the study, enzalutamide was approved by the US Food and Drug Administration for use only in patients with mCRPC (approval for post-chemotherapy mCRPC was on August 31, 2012, and chemotherapy-naïve mCRPC on September 10, 2014) [29, 30], though its indication was extended on July 13, 2018 (after the study period), to CRPC, which included nmCRPC [31]. This extended indication was based on the prolonged metastasis-free survival observed in the PROSPER study [32]. Prior real-world studies have relied on documentation of prior/no chemotherapy in their database to stratify patients [11, 26, 33, 34], and a similar approach has been followed in the current study to include chemotherapy-naïve patients. Evidence also suggests that in the US, specialists in the urology setting prefer to treat their patients with a non-chemotherapy option and move them to the oncology setting for chemotherapy, suggesting that this study population most likely consists of chemotherapy-naïve patients [35]. To reduce misclassification, we excluded patients with a documented history of chemotherapy treatment. However, due to missing data, including undocumented metastatic status for ~70% of patients and nearly 7% of patients without documentation of prior treatment with hormonal therapy before index, patients’ mCRPC status could not be confirmed. The challenges with missing data are common while using real-world databases. In a previous real-world study conducted to evaluate the PSA response in Black and White patients treated with abiraterone acetate for mCRPC, data for metastatic status were available for 76% of Black and 81% of White patients, indicating that about 20% of patients had missing metastatic status [21]. Another real-world study on treatment outcomes of patients with advanced PC also reported missing metastatic diagnoses and sites of metastases as a study limitation [34]. Further, some patients in this study population may have been misclassified as castration-resistant when they had non-castrate levels of testosterone at some points during follow-up due to the infrequent rate of assessing testosterone levels in the real world and the limitation of the data source. Moreover, previous real-world studies evaluating treatment response and outcomes in patients with mCRPC have not reported testosterone baseline levels or included castrate levels of testosterone as an exclusion criterion [21–23, 26, 27]. An intermediate endpoint in this study, cPFS, was used instead of PFS, as the EMR database used in this study does not have complete documentation on the status of metastasis. Considering the disparities in PC between races and the potential for referral to an oncologist for cancer management that may bias estimates of outcome rates, we assessed PSA testing frequency during follow-up, enzalutamide treatment duration, and duration of follow-up but found no meaningful difference by race (Supplementary Fig. 1). Further, because the data in the database consist only of urologist office data and censoring may occur when patients move from urologists to oncologists, the longitudinality of the data is limited. OS was not assessed in this study since the data source from the urology clinical EMR setting may not have captured data related to deaths, as patients may have transitioned to an oncology clinical setting and were therefore lost to follow-up. Whether these results apply to patients outside the IQSS urology EMR database requires further study.

## Conclusions

Black and White men with presumed chemotherapy-naïve mCPRC had similar PSA responses when treated with enzalutamide. However, Black men may have better cPFS than White men during enzalutamide treatment. This study reinforces the efficacy of enzalutamide for the treatment of patients with presumed chemotherapy-naïve mCRPC, irrespective of race. Outcomes of this study are consistent with those of other real-world studies that assessed treatment outcomes in Black and White men with PC treated with NHTs. Further research is warranted to validate these findings.

## Supplementary information

Supplementary material

## Data Availability

Researchers may request access to anonymized participant level data, trial level data and protocols from Astellas sponsored clinical trials at www.clinicalstudydatarequest.com. For the Astellas criteria on data sharing see: https://clinicalstudydatarequest.com/Study-Sponsors/Study-Sponsors-Astellas.aspx.
