# Supplementary material for: Prostate-specific antigen response and clinical progression-free survival in Black and White men with chemotherapy-naïve metastatic castration-resistant prostate cancer treated with enzalutamide in a real-world setting
Source: Prostate Cancer Prostatic Dis. 2022 Dec 14;26(3):523–30. doi: 10.1038/s41391-022-00622-6 (PMC10449620; doi:10.1038/s41391-022-00622-6)
Supplement: Supplementary file 1 — Supplementary material [file 41391_2022_622_MOESM1_ESM.pdf]

Supplementary material

Supplementary Fig. 1 Post-index PSA test count

Supplementary Fig. 2 Kaplan-Meier curves for ≥75% PSA decline

Supplementary Table 1 Summary of multivariable Cox analysis for association of race with PSA decline from baseline

Supplementary Table 2 Summary of multivariable Cox analysis for association of race with cPFS

Supplementary Table 3 Summary of multivariable Cox analysis for association of race with 12-month cPFS

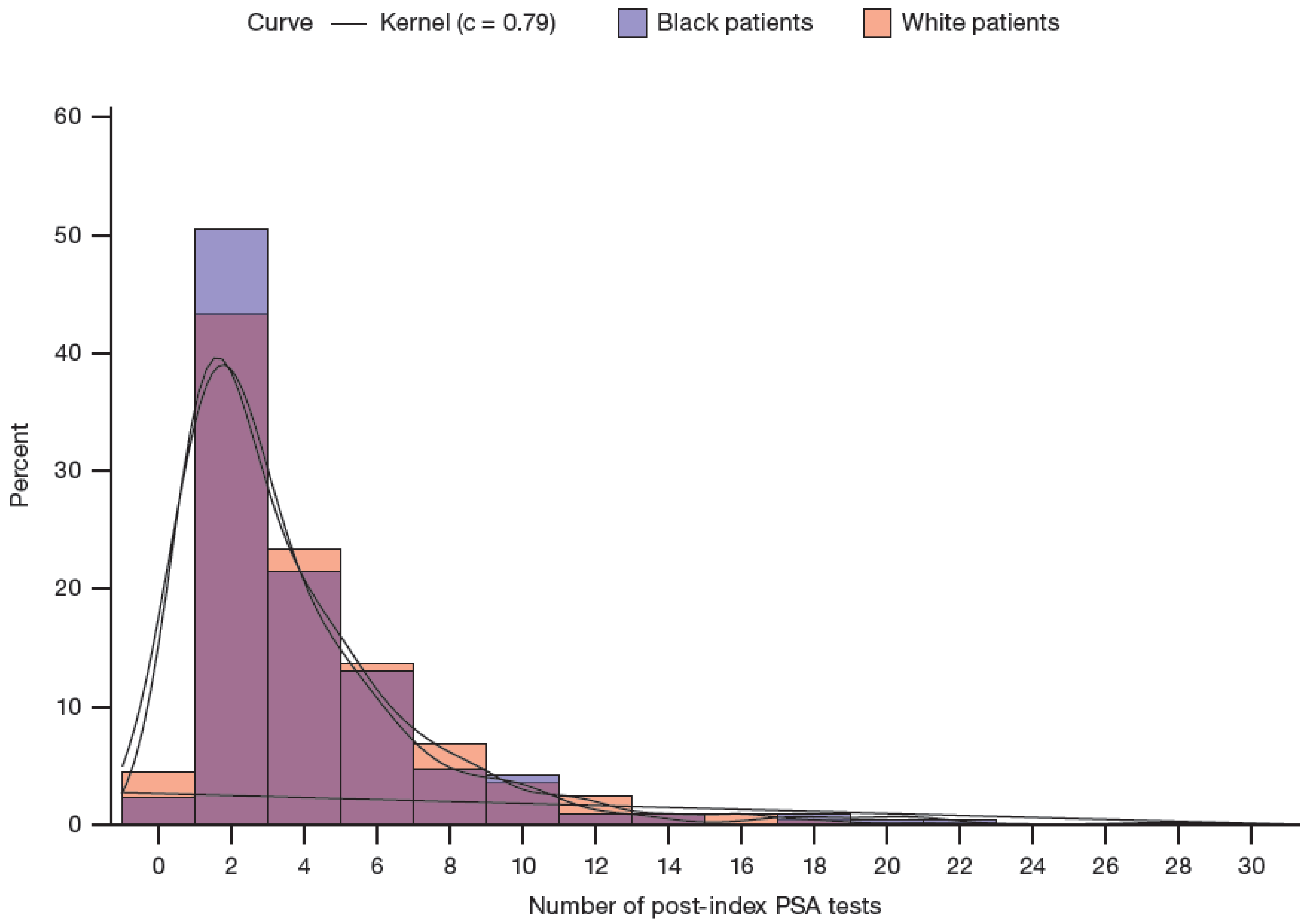

Curve
Kernel (c = 0.79)
Black patients
White patients
Percent
60
50
40
30
20
10
0
0
2
4
6
8
10
12
14
16
18
20
22
24
26
28
30
Number of post-index PSA tests

**Post-index PSA tests**	**Black patients, n (%) (n = 214)**	**White patients, n (%) (n = 1332)**	***P* value**
**0**[a]	5 (2.3)	59 (4.4)	0.5874
**1**	55 (25.7)	300 (22.5)	
**2**	53 (25.7)	277 (20.8)	
**3**	28 (13.1)	178 (13.4)	
**4**	18 (8.4)	133 (10.0)	
**5**	16 (7.5)	108 (8.1)	
**6**	12 (5.6)	73 (5.5)	
**7+**	27 (12.6)	204 (15.3)	
**Mean (SD)**	3.52 (3.35)	3.70 (3.34)	0.3290
**Median (IQR)**	2.00 (1.00–5.00)	3.00 (1.00–5.00)	

Supplementary Fig. 1 Post-index PSA test count. [a]PSA tests for patients who died were not included in the post-index period. *PSA* prostate-specific antigen.

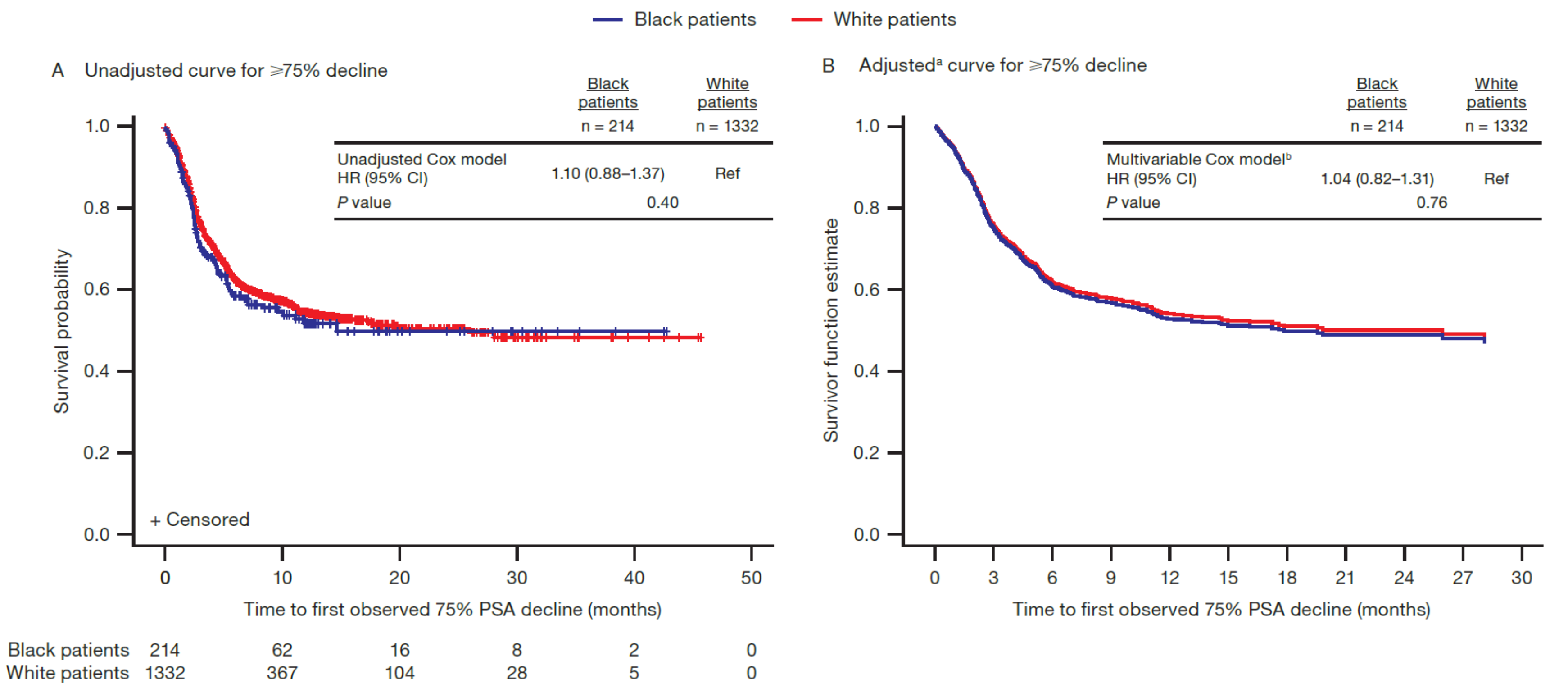

Supplementary Fig. 2 Kaplan-Meier curves for ≥75% PSA decline. [a]Since adjusted curves graph only events, the population is followed up only until the last event. Any values censored after the last event cannot be displayed on adjusted KM curves. [b]Multivariable Cox proportional HR adjusted for baseline covariates of age, region, marital status, year of enzalutamide initiation, CCI, baseline log PSA, pre-index treatments, and documentation of metastasis. *CCI* Charlson Comorbidity Index, *KM* Kaplan-Meier, *PSA* prostate-specific antigen, *Ref* reference.

Supplementary Table 1 Summary of multivariable Cox analysis for association of race with PSA decline from baseline PSA

	≥50% PSA decline			≥75% PSA decline			≥90% PSA decline		
**Baseline characteristic**	**HR**	**95% CI**	***P* value**	**HR**	**95% CI**	***P* value**	**HR**	**95% CI**	***P* value**
**Race**									
White	Ref	- -	-	Ref	- -	-	Ref	- -	-
Black	1.02	[0.83, 1.25]	0.8822	1.04	[0.82, 1.31]	0.7638	1.23	[0.93, 1.62]	0.1435
**Age group**									
40–59	Ref	- -	-	Ref	- -	-	Ref	- -	-
60–79	0.95	[0.67, 1.35]	0.7736	0.87	[0.58, 1.29]	0.4831	0.74	[0.47, 1.19]	0.2114
≥80	0.86	[0.60, 1.25]	0.4387	0.74	[0.49, 1.13]	0.1636	0.63	[0.38, 1.03]	0.0668
**Geographic region**									
South	Ref	- -	-	Ref	- -	-	Ref	- -	-
Midwest	1.14	[0.96, 1.35]	0.1329	1.13	[0.93, 1.38]	0.2091	1.29	[1.02, 1.64]	0.0358
Northeast	0.90	[0.74, 1.09]	0.2836	0.96	[0.76, 1.20]	0.6963	0.98	[0.74, 1.31]	0.9020
West	1.01	[0.79, 1.29]	0.9461	1.06	[0.80, 1.41]	0.6726	1.13	[0.79, 1.60]	0.5074
Unknown	1.60	[0.65, 3.91]	0.3062	1.41	[0.52, 3.85]	0.4982	1.62	[0.51, 5.19]	0.4138

**Marital status**												
Single	Ref	-	-	-	Ref	-	-	-	Ref	-	-	-
Married	0.97	[0.76,	1.24]	0.8091	1.02	[0.77,	1.37]	0.8771	1.01	[0.71,	1.43]	0.9642
Divorced	1.30	[0.89,	1.88]	0.1716	1.32	[0.86,	2.02]	0.1997	1.21	[0.72,	2.05]	0.4699
Separated	0.99	[0.70,	1.40]	0.9657	1.15	[0.78,	1.70]	0.4901	1.28	[0.80,	2.03]	0.2999
Unknown	1.23	[0.89,	1.69]	0.2080	1.18	[0.81,	1.71]	0.3894	0.96	[0.60,	1.53]	0.8616
**Year of index therapy initiation**												
2014	Ref	-	-	-	Ref	-	-	-	Ref	-	-	-
2015	0.91	[0.69,	1.20]	0.5014	1.05	[0.76,	1.46]	0.7675	1.23	[0.80,	1.88]	0.3430
2016	0.85	[0.64,	1.12]	0.2381	0.96	[0.69,	1.33]	0.7981	1.11	[0.73,	1.70]	0.6230
2017	1.00	[0.76,	1.31]	0.9744	1.11	[0.80,	1.54]	0.5279	1.40	[0.92,	2.14]	0.1154
2018	1.12	[0.70,	1.82]	0.6321	1.14	[0.64,	2.02]	0.6623	1.09	[0.49,	2.43]	0.8279
**CCI score**	0.98	[0.92,	1.05]	0.5653	1.01	[0.94,	1.09]	0.8079	1.02	[0.93,	1.12]	0.6386
**Log baseline PSA**	1.08	[1.04,	1.12]	<0.0001	1.11	[1.06,	1.15]	<0.0001	1.14	[1.08,	1.20]	<0.0001
**Pre-index therapies**												
Antiandrogen therapy	0.87	[0.75,	1.01]	0.0575	0.71	[0.60,	0.84]	<0.0001	0.74	[0.60,	0.92]	0.0055
Pain management therapy	0.93	[0.79,	1.09]	0.3651	0.84	[0.70,	1.01]	0.0704	0.75	[0.59,	0.95]	0.0158
Bone-targeting agents	0.90	[0.78,	1.05]	0.1907	0.95	[0.80,	1.13]	0.5602	0.91	[0.74,	1.13]	0.4073

**Site of metastasis**												
Undocumented	Ref	-	-	-	Ref	-	-	-	Ref	-	-	-
Documented	1.23	[1.05,	1.45]	0.0104	1.06	[0.88,	1.28]	0.5252	0.88	[0.69,	1.11]	0.2830

CCI Charlson Comorbidity Index, *PSA* prostate-specific antigen, *Ref* reference.

Supplementary Table 2 Summary of multivariable Cox analysis for association of race with cPFS

Baseline characteristic	HR	95% CI		*P* value
**Race**				
White	Ref	-	-	-
Black	0.82	[0.68,	0.98]	0.0290
**Age group**				
40–59	Ref	-	-	-
60–79	0.93	[0.68,	1.28]	0.6539
≥80	1.02	[0.74,	1.42]	0.8913
**Geographic region**				
South	Ref	-	-	-
Midwest	1.00	[0.87,	1.16]	0.9955
Northeast	1.13	[0.96,	1.32]	0.1558
West	0.90	[0.73,	1.11]	0.3256
Unknown	2.08	[1.03,	4.24]	0.0424
**Marital status**				
Single	Ref	-	-	-
Married	1.07	[0.87,	1.32]	0.5418
Divorced	0.88	[0.63,	1.24]	0.4566
Separated	0.99	[0.75,	1.32]	0.9577
Unknown	0.79	[0.60,	1.05]	0.1055

**Year of index therapy initiation**				
2014	Ref	-	-	-
2015	0.95	[0.76,	1.18]	0.6184
2016	0.91	[0.73,	1.13]	0.3964
2017	0.85	[0.67,	1.07]	0.1564
2018	1.11	[0.67,	1.84]	0.6950
**CCI score**	1.01	[0.96,	1.07]	0.6336
**Log baseline PSA**	1.04	[1.01,	1.07]	0.0094
**Pre-index therapies**				
Antiandrogen therapy	1.01	[0.89,	1.14]	0.9390
Pain management therapy	1.23	[1.08,	1.39]	0.0019
Bone-targeting agents	1.06	[0.93,	1.20]	0.3743
**Site of metastasis**				
Undocumented	Ref	-	-	-
Documented	1.28	[1.12,	1.46]	0.0003

CCI Charlson comorbidity index, *cPFS* clinical progression-free survival, *PSA* prostate-specific antigen, *Ref* reference.

Supplementary Table 3 Summary of multivariable Cox analysis for association of race with 12-month cPFS

Baseline characteristics	HR	95% CI		*P* value
**Race**				
White	Ref	-	-	-
Black	0.84	[0.68,	1.03]	0.0853
**Age group**				
40-59	Ref	-	-	-
60-79	1.05	[0.73,	1.51]	0.8076
≥80	1.22	[0.84,	1.78]	0.2972
**Geographic region**				
South	Ref	-	-	-
Midwest	0.98	[0.84,	1.15]	0.8219
Northeast	1.14	[0.95,	1.36]	0.1612
West	0.95	[0.76,	1.19]	0.6557
Unknown	1.66	[0.73,	3.74]	0.2263
**Marital status**				
Single	Ref	-	-	-
Married	1.10	[0.87,	1.39]	0.4117
Divorced	0.86	[0.59,	1.27]	0.4488
Separated	1.08	[0.79,	1.48]	0.6372
Unknown	0.91	[0.66,	1.24]	0.5393

**Year of index therapy initiation**				
2014	Ref	-	-	-
2015	1.12	[0.86,	1.44]	0.3971
2016	0.98	[0.76,	1.27]	0.8813
2017	0.99	[0.76,	1.29]	0.9511
2018	1.27	[0.75,	2.13]	0.3747
**CCI score**	1.01	[0.95,	1.07]	0.7006
**Log baseline PSA**	1.04	[1.01,	1.08]	0.0105
**Pre-index therapies**				
Antiandrogen therapy	1.00	[0.87,	1.15]	0.9732
Pain management therapy	1.30	[1.13,	1.49]	0.0002
Bone-targeting agents	1.06	[0.92,	1.22]	0.4280
**Site of metastasis**				
Undocumented	Ref	-	-	-
Documented	1.35	[1.17,	1.56]	<0.0001

CCI Charlson comorbidity index, *cPFS* clinical progression-free survival, *PSA* prostate-specific antigen, *Ref* reference.
